# More Than a Wrinkle: The Burden of Franck’s Sign

**DOI:** 10.1007/s11606-026-10437-z

**Published:** 2026-04-22

**Authors:** Angelica Lee

**Affiliations:** Department of Neurology and Rehabilitation Medicine, School of Medicine, Uniformed Services University of the Health Sciences (USUHS), 4301 Jones Bridge Road, Bethesda, MD 20814 USA

I sink into my recliner and scroll, looking for a show—any distraction to offer a brief escape. I finally settle on a show with a very well-known actor and familiar face, an iconic actor I’ve admired since I was a kid—he’s funny, with a charisma that makes you feel like you know him, a parasocial friend in a time of profound isolation. He is the perfect guide to lead me away from my own thoughts.

In the first episode, a dramatic scene unfolds in a motel room. As his character, who is a bounty hunter, tries to catch a bad guy, he is tackled. The camera zooms in on his face before the scene reaches its climax. It was meant to be a moment of high drama, a cliffhanger to pull the audience in. For me, it does something else entirely. My clinical eye, a part of me I thought was dormant, catches something familiar. This is a man I know to be otherwise fit and healthy, though I have no idea about his lifestyle or smoking history. But there it is, clear as day: Frank’s sign.

In that instant, the comfort of my distraction vanished, replaced by the familiar, involuntary hum of clinical reasoning. It’s a feeling every physician knows—a switch that flips, pulling you from the role of passive observer to active analyst. The wall I had carefully constructed between my private life and my professional identity crumbled. It is a sign so common that I have observed it in friends, co-workers, and now, celebrities on the TV screen. The world, it seems, is my clinic, and I am always on-call.

Frank’s sign, also known as the diagonal earlobe crease (DELC), has been a subject of medical discussion and study for over 50 years^1^. It refers to a deep crease or wrinkle that extends diagonally from the tragus across the earlobe^2^. It was first reported in 1973 by Dr. Sanders T. Frank, who observed the sign in patients suffering from angina and coronary artery disease (CAD). Since then, its presence has been associated with an increased risk of cardiovascular events^3^. While not a definitive diagnostic tool, it remains a compelling clue in a much larger puzzle.

As the actor continued his scene, unaware of the differential diagnosis I had just constructed from my recliner, I was left to wonder—at what point does the clinician’s gaze become a burden? When, if ever, should I tell a friend or approach a co-worker about this sign? Would my concern be seen as a caring gesture, or would it create an unnecessary moment of anxiety? Should I compartmentalize my knowledge, and move on, or does silence constitute a quiet betrayal?

The scientific literature offers a compelling case for paying attention. Early controlled studies compared hundreds of patients with significant coronary heart disease to control populations and found the sign present in over 70% of the coronary group, compared to only 30% of the controls^4^. Subsequent research has found this association to be independent of traditional risk factors such as age, smoking, or cholesterol. In a prospective analysis of patients undergoing coronary cineangiography, the earlobe crease was one of the few physical markers that correlated strongly with demonstrable disease^5^.

Yet, there is a crucial word of caution against overinterpretation. The literature emphasizes that the presence of DELC should be regarded as a “physical marker,” rather than a diagnostic test^5^. An individual with the crease is “at risk,” but it does not definitively mean they have CAD. This distinction sits at the heart of my dilemma. The sign is an alarm bell, but what is the protocol for ringing an alarm bell in a social setting?

As a neurologist, my fascination is rooted in the intrinsic connection between the heart and the brain. I admit, I am obsessed with Frank’s sign because CAD may affect every organ, particularly the one I have sworn to protect: the brain. An ischemic stroke, at its core, is often a plumbing problem—a disease of blood vessels that manifests with devastating neurological consequences. Research has confirmed that nearly 79% of acute ischemic stroke patients exhibit the sign, an association that is even stronger in those under 60^6^.

In my practice, this knowledge translates into a clear duty to counsel my patients. I constantly struggle with the ethical tightrope walk. On one side is the principle of beneficence—the duty to act for the good of others. On the other is non-maleficence—the duty to do no harm, which includes the psychological harm of causing undue anxiety over a statistical risk. And what of their autonomy, their right *not* to know?

I imagine the scenarios. Do I pull a colleague aside after a meeting? Do I risk ruining a dinner party with friends by turning a lighthearted conversation toward cardiovascular health? The potential for being dismissed as “the morbid friend” who can’t turn off her clinical brain is immense. Yet, if something were to happen to them, would my silence haunt me? The weight of that “what if” can be heavier than any social awkwardness.

Medical education is a permanent lens. Once we are trained to see patterns, there is no going back. Our training filters every interaction, every observation in the clinic, every face on a television screen. The line between a physician’s duty and a friend’s discretion is not just blurry; at times, it feels nonexistent.

In the end, I think, there is no single answer, no universal protocol for these moments. The decision to speak must be weighed against the relationship, the context, and the potential for genuine benefit versus harm. But the observation itself is unavoidable. The actor on the screen finished his scene and moved on, forever unaware of the diagnosis rendered from my recliner miles away. On the other hand, I failed miserably in my quest for distraction. Instead, I was reminded of the profound and often burdensome privilege of being a physician.
